# Fighting against kidney diseases with small interfering RNA: opportunities and challenges

**DOI:** 10.1186/s12967-015-0387-2

**Published:** 2015-02-01

**Authors:** Cheng Yang, Chao Zhang, Zitong Zhao, Tongyu Zhu, Bin Yang

**Affiliations:** Department of Urology, Zhongshan Hospital, Fudan University, Shanghai, China; Shanghai Key Laboratory of Organ Transplantation, Shanghai, China; Transplant Group, Department of Infection, Immunity and Inflammation, University Hospitals of Leicester, University of Leicester, Leicester, UK; Department of Nephrology, Affiliated Hospital of Nantong University, Nantong, China; Basic Medical Research Centre, Medical School of Nantong University, Nantong, China

**Keywords:** Small interfering RNA, Kidney disease, Delivery, Off-target effect and compensative response

## Abstract

The significant improvements in siRNA therapy have been achieved, which have great potential applications in humans. The kidney is a comparatively easy target organ of siRNA therapy due to its unique structural and functional characteristics. Here, we reviewed recent achievements in siRNA design, delivery and application with focuses on kidney diseases, in particular kidney transplant-related injuries. In addition, the strategy for increasing serum stability and immune tolerance of siRNA was also discussed. At last, the future challenges of siRNA therapy including organ/tissue/cell-specific delivery and time-controlled silence, as well as selecting therapeutic targets, were addressed as well.

## Introduction

RNA interference (RNAi) is a highly conserved biological phenomenon in all eukaryotes, including renal cells. In the late 1990s, due to the development of molecular biology and genetics, the biological understanding of RNA evolved from simply an intermediate between DNA and protein to a dynamic and versatile regulator that functions in genes and cells in all living organisms. In 1998, as a milestone, Fire *et al*. injected a few molecules of double-stranded RNA (dsRNA) into *Caenorhabditis elegans* and found that dsRNAs could specifically interfere with the protein expression of an endogenous gene [1]. This molecule was named small interfering RNA (siRNA) that mediates RNAi [2-4]. siRNA is able to recognize and degrade a homologous host mRNA. Therefore, the gene from which the mRNA is transcribed is silenced, which is referred to as post-transcriptional gene silencing [5,6].

Although RNAi naturally exists, synthetic artificial siRNA exerts similar effects as natural endogenous microRNA (miRNA). Both sense and antisense strands of siRNA can be synthesized separately and annealed to form double stranded siRNA duplexes *in vitro.* After the siRNA is delivered into the cytoplasm, the artificial siRNA silences the target gene using similar biological processes as endogenous miRNA. Since the introduction of 21-nucleotide artificial siRNAs that triggered gene silencing in mammalian cells [7], synthetic siRNA has generated much interest in biomedical research, in which the kidney is one of important key players. siRNA as a strategic molecule has been highly expected in the field of innovative therapy. Because siRNA is highly efficient at gene silencing, it is possible to develop specific siRNA-based drugs that could target any genes, including those that have no known pharmacological antagonists or inhibitors. Different types of synthetic siRNA have been tested for their efficacy in various disease models, including cancer [8,9], autoimmune disorders [10], cardiovascular injuries [11,12], and organ transplantation [13,14], including native and transplanted kidney injuries [15].

As siRNA is a posttranscriptional regulator, it must first be absorbed into the target cells. Therefore, the kidney could be an excellent target organ for siRNA therapy because it benefits from rapid, vast blood flow physically and subsequent glomerular filtration and tubular absorption. In fact, systemic administration of siRNA leads to rapid uptake by the kidney, yielding a significant decrease of target protein expression [15]. Consequently, RNAi by siRNA has advantages for the treatment of renal diseases due to the unique urological system. In addition, the preservation of donor kidneys before transplantation also provides a suitable time window for the intervention of siRNA. Therefore, we performed a series of experiments using naked caspase-3 siRNA to investigate its efficacy, off-target effects and compensative responses in *in vitro*, *ex vivo* and *in vivo* models of transplant-related renal injuries.

In this review, we highlighted the design and delivery of siRNA, its therapeutic effects, off-target responses and systematic compensations, as well as potential challenges, with a focus on kidney diseases, including ongoing clinical trials.

## Current principle of siRNA design

The design of potent siRNAs has been greatly improved over the past decade. The basic criteria for choosing siRNAs includes the consideration of thermodynamic stability, internal repeats, immunostimulatory motifs, such as GC content, secondary structure, base preference at specific positions in the sense strand, and appropriate length [16].

Chemical modifications significantly enhance the stability and uptake of naked siRNAs. Importantly, siRNAs can be directly modified without crippling the silencing ability. Chemical modifications have been rigorously investigated for virtually every part of siRNA molecules, from the termini and backbone to the sugars and bases, with the goal of engineering siRNA to prolong half-life and increase cellular uptake. The most common chemical modification involves modifying the sugar moiety. For example, the incorporation of 2’-fluoro (2’-F), −O methyl, −halogen, −amine, or -deoxy can significantly increase the stability of siRNA in serum. Locked nucleic acid (LNA) has been also applied to modify siRNA. The commonly used LNA contains a methylene bridge connecting the 2’-oxygen with the 4’-carbon of the ribose ring. This bridge locks the ribose ring in the 3’-endo conformation characteristic of RNA [17]. Additionally, recent studies, including ours [18], have proven the efficacy of LNA modified siRNA in terms of prolonged half-life in serum, but without detectable adverse effects, suggesting that the natural RNAi machinery could accommodate a certain degree of alterations in the chemical structure of siRNAs [19].

## siRNA delivery

The biggest obstacle faced by siRNA therapies is the *in vivo* delivery of genetic materials. The virus-based delivery system, while efficient, may be fatally flawed due to raised safety concerns, such as inducing mutations and triggering immunogenic and inflammatory responses [20]. Therefore, extensive research had been performed to develop efficacious non-viral delivery systems, including direct chemical modification of siRNA (as described above) and/or optimization of delivery materials, such as liposome formulation, nanoparticle conjugation and antibodies that target cellular moieties [20].

To date, studies on synthetic siRNA therapy have been performed in a variety of cell culture and rodent models [21] that produced exciting results and were cost effective, but failed to faithfully mimic human diseases. Therefore, large animal models, such as porcine models, are indispensable to compensate for the limitations of rodent models due to their greater similarity to human beings. The investigations on siRNA conducted in our laboratory have reflected this trend in the field [14,18,22].

### In vitro delivery

Cell culture is an important model for investigating the cellular and molecular mechanisms of diseases. Lentivirus vectors and liposomes are widely applied for the transduction of siRNA into different types of cultured renal cells. For example, silencing *ccr2* using its siRNA delivered by a lentivirus significantly ameliorated MCP-1 induced podocyte apoptosis under diabetic conditions [23]. Similarly, degradation of FoxO1 by transfecting its siRNA via lentiviral vectors overrode the limited cell cycle and stimulated proliferation of glomerular mesangial cells [24]. In our previous study, we transfected porcine proximal tubular cells (LLC-PK1) with synthetic caspase-3 siRNA using a cationic lipid-based transfection reagent. The caspase-3 siRNA inhibited apoptosis and inflammation in LLC-PK1 cells that were subjected to hydrogen peroxide stimulation [25].

### Ex vivo delivery

In addition to *in vitro* delivery of siRNA, *ex vivo*/*in vivo* siRNA delivery to target organs is an indispensable step before its clinical application. If it were directly delivered into the kidneys, siRNA could obtain higher local concentrations, which would result in improved gene silencing efficacy. During kidney transplantation, *ex vivo* local delivery of siRNA into the donor kidney is feasible because it could be facilitated by the unique structure of the kidney and the characteristics of kidney transplantation. Recently, we utilized an *ex vivo* isolated porcine kidney reperfusion system to assess the efficacy of naked caspase-3 siRNA. The caspase-3 siRNA was directly infused into the renal artery (locally) and autologous blood perfusate (mimic systemic delivery) before 24-hour cold storage (CS), followed by a further reperfusion for 3 hours. The results demonstrated that the caspase-3 siRNA improved ischemic reperfusion (IR) injury with reduced caspase-3 expression and apoptosis, better renal oxygenation and acid–base homeostasis [22]. These promising proof-of-principle observations provide valuable guidance for further development before siRNA clinical practice.

### In vivo local delivery

Based on the anatomical and physiological characteristics of the kidney, local delivery can be achieved through several routes: (1) renal artery: first targeting the glomeruli or tubules [26,27]; (2) renal vein: predominately targeting tubulointerstitium [28]; (3) intra urethral: administered into the renal pelvis and interstitium [29]; and (4) sub-capsular administration: achieves intraparenchymal silencing [30]. Due to the rich blood flow through the glomeruli, siRNA injection via the renal artery followed by electroporation could silence specific genes in the glomeruli, such as TGF-β 1, which subsequently ameliorates matrix expansion in an experimental glomerulonephritis model [26]. However, acute kidney injury (AKI), such as IR injury, is usually characterized by tubular apoptosis and inflammatory infiltration in the tubulointerstitium, which should be suitable for local siRNA delivery via any above mentioned method.

It has been revealed that an injection of a single-dose Fas siRNA through the renal vein post ischemia provided a survival advantage in a murine IR model, which was due to the anti-apoptosis and anti-inflammation effects of the Fas siRNA [28]. Unilateral ureteral obstruction (UUO) is a well-established model for tubulointerstitial fibrosis. Xia et al. injected the siRNA of heat shock protein 47 once via the ureter at the time of UUO preparation, leading to significantly reduced fibrosis-related protein expression and a remarkable alleviation of the accompanying interstitial fibrosis [29]. Sub-capsular administration is still used in some experiments due to its unique advantages, although it requires an invasive procedure and has limitations in clinical practice. Cuevas et al. reported that an infusion of DJ-1 (an antioxidant) specific siRNA into the sub-capsule silenced DJ-1 expression in the renal cortex and increased ROS production [30].

### In vivo systemic delivery

Systemic delivery is a common and convenient clinical practice, although current clinical trials using siRNAs are almost directly administered to the target site, such as the nostril, eye and lung, thereby avoiding the complexity of systemic delivery [31]. The most common method of systemic siRNA delivery is a hydrodynamic intravenous injection with hydraulic pressure to assist siRNA cell entry. However, the pharmacokinetic metabolism of siRNA is more complicated during systemic delivery because siRNAs can be rapidly degraded by nucleases in the serum and cleared by the kidney and liver. To enhance the *in vivo* efficacy of siRNA treatment, a variety of approaches have been attempted for both siRNA itself and delivery techniques [30-36], as mentioned above and discussed later.

Due to its anatomical and physiological characteristics, the kidney is the most preferable target organ of systemic siRNA administration. siRNA access to the kidney is thought to be dependent on the filtration and reabsorption functions of the kidney. Proximal tubule cells (PTCs) are the primary site for rapid and extensive endocytic uptake of siRNA within the kidney following glomerular filtration. In an AKI model, naked synthetic siRNA targeting p53 that was intravenously injected 4 hours after renal ischemic injury significantly reduced upregulated p53 expression and protected both the PTCs and kidneys [37]. In another study performed by Zheng et al., siRNA was systematically injected to target complement 3 (C3) and caspase-3 in a murine renal IR injury model. The results showed that the level of serum creatinine and blood urea nitrogen was significantly decreased in the siRNA-treated mice [38]. These studies highlighted the potential feasibility of systemically delivered siRNA for the treatment of kidney diseases in future clinical practice.

## siRNA therapeutic efficacy

To date, siRNA therapy has been successfully applied for acute and chronic kidney diseases, as well as renal tumors.

### Acute kidney injury

AKI is a common complication of hospitalization that has a mortality rate as high as 30-50% [39,40]. IR injury is the primary cause of AKI, particularly during kidney transplantation, in which the kidney is exposed to hypoxia and experiences a series of oxidative, inflammatory and apoptotic responses [41,42]. Consequently, specific siRNAs targeting critical molecules that are involved in the processes of oxidation, inflammation and apoptosis have been developed.

Caspase-3, which mediates apoptosis and inflammation, is upregulated by IR injury. Multiple pharmacological interventions against caspase-3, including enzyme inhibitors and genetic modification, have been investigated. In recent years, our group studied the delivery and efficacy of caspase-3 siRNA in *in vitro*, *ex vivo* and *in vivo* porcine models. The synthetic caspase-3 siRNA was initially tested in porcine PTCs, with or without hydrogen peroxide (H_2_O_2_) stimulation. Apoptotic cells and activated IL-1β protein expression were significantly reduced by the caspase-3 siRNA, with improved cell viability [25]. This outcome led to siRNA application in an isolated organ perfusion system, as described above, and the efficacy of caspase-3 siRNA was further proven [22].

**Figure 1:**
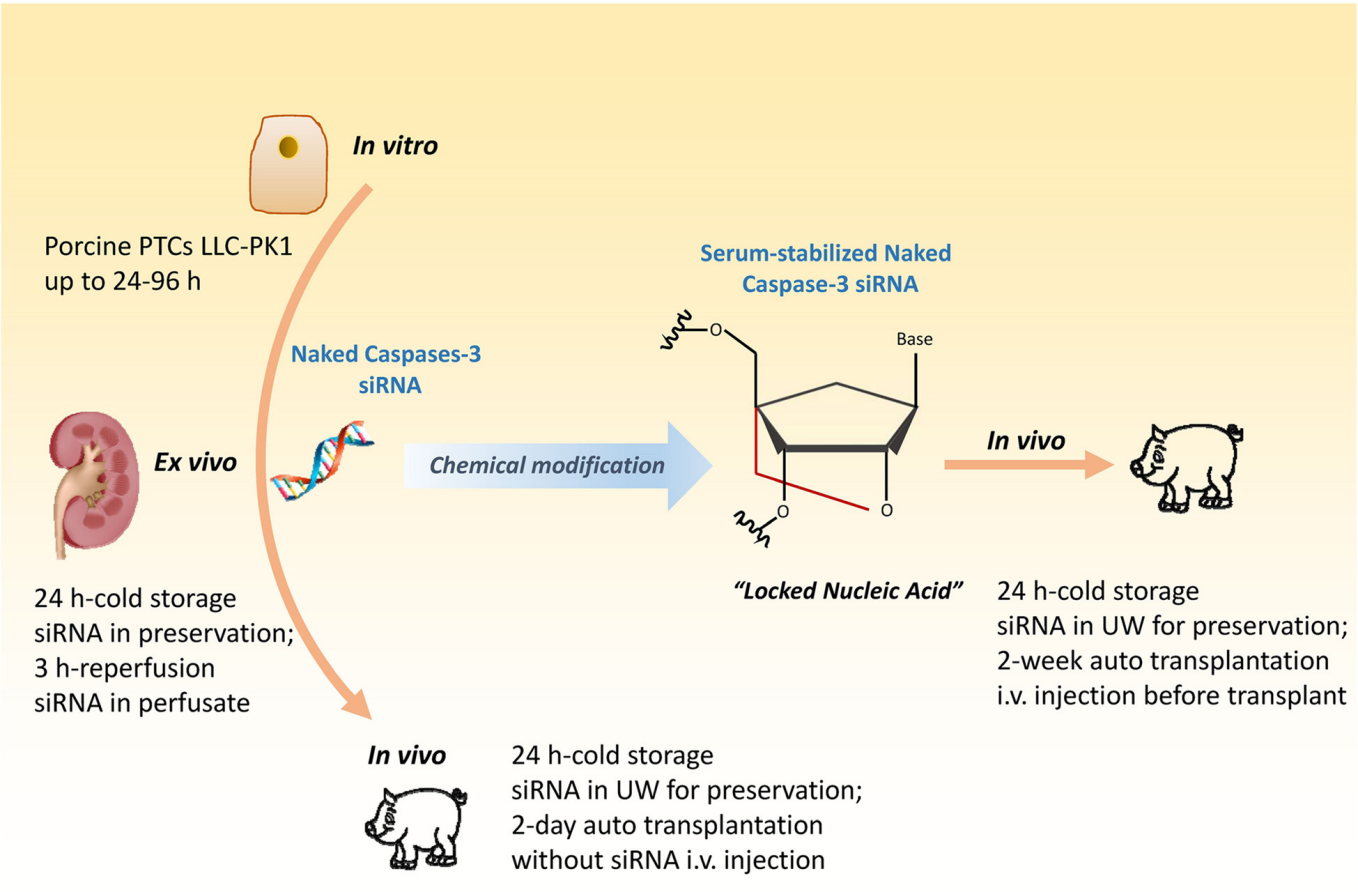
**Schematic drawing showed a series of studies using caspase-3 siRNA.** The caspase-3 siRNA was first used to protect porcine renal tubular epithelia cells against hydrogen peroxide-induced injury. The renoprotection of naked caspase-3 siRNA with the same sequences was further validated in a porcine *ex vivo* isolated reperfusion model, then shown that the siRNA was effective for cold preservation, but not in auto-transplanted kidneys without systematic siRNA treatment. Finally, the modified siRNA of caspase-3 via locked nucleic acid stabilized the siRNA in serum, and significantly protected auto-transplanted kidneys.

We then used naked caspase-3 siRNA in a porcine kidney auto-transplant model for the first time. The left kidney was retrieved from mini pigs and was infused with University of Wisconsin solution, with or without 0.3 mg naked caspase-3 siRNA, via the renal artery, which was followed by renal artery and renal vein clamping for 24-hour cold storage (CS, mimicking donor kidney preservation before transportation in clinic). After right nephrectomy, the left kidney was auto-transplanted into the right nephridial pit for 48 hours without systemic siRNA treatment (Figure 1). The expression of caspase-3 mRNA and active caspase-3 protein, as well as its precursor, was downregulated by siRNA in the post-CS kidney. In the siRNA preserved post-transplant kidney, however, caspase-3 precursor was further decreased while caspase-3 mRNA and its activated subunits were upregulated, which resulted in increased apoptosis and inflammation. This study indicated that the naked caspase-3 siRNA was effective for cold preservation but was not effective at protecting post-transplant kidneys, which may be due to systemic compensative responses overcoming local effects. Therefore, to overcome the systemic response and prolong the therapeutic time window, we subsequently utilized a novel, serum stable caspase-3 siRNA, both locally as before and systemically via a pre-transplantation intravenous injection, and observed the animals for up to 2 weeks post-transplantation. The effectiveness of the novel caspase-3 siRNA was confirmed by downregulated caspase-3 mRNA and protein in the post-CS and/or post-transplant kidneys, as well as reduced apoptosis and inflammation. More importantly, renal function, associated with active caspase-3, HMGB1, apoptosis, inflammation and tubulointerstitial damage, was improved by this novel, serum stable caspase-3 siRNA [18].

p53, another pivotal protein in the apoptotic pathway, has been identified as a mediator of transcriptional responses to IR injury [43-45]. Molitoris et al. revealed that intravenously injected p53 siRNA attenuated ischemic and cisplatin-induced AKI [37]. Fujino et al. also tested the efficacy of transarterial administration of siRNA that targeted p53. p53 siRNA injected into the left renal artery immediately after ischemia improved tubular injury and downregulated GSK-3β expression [46]. In a diabetic mouse model, p53 inhibition by siRNA also reduced ischemic AKI [47].

Silencing other important transcription factors or immunity related receptors using siRNAs have also been studied. Renal IR injury and inflammation are related to postsurgical healing and both processes can be influenced by Toll-like receptor (TLR) signals. Effective TLR9 silencing by siRNA decreases renal cell apoptosis, mitigates AKI severity, and increases the mice survival [48]. NF-κB, a pro-inflammatory transcription factor induced by TLR and other signals, plays a key role in AKI. NF-κB activation depends on the activation of the inhibitor of κB kinase β (IKKβ). Wan et al. demonstrated that silencing IKKβ using siRNA diminished inflammation and protected the kidneys against IR injury [27]*.* These studies clearly demonstrate the therapeutic potential of siRNA-induced silencing of key AKI mediators, which are activated and involved in the pathways of apoptosis, inflammation, immunity, etc.

### Chronic renal disease

Glomerulonephritis, resulting from multiple factors, is the most common primary disease leading to chronic kidney dysfunction. Interventions based on siRNA therapy for glomerulonephritis are promising, although a tissue-specific regimen has not been established. Shimizu et al. utilized the electrostatic interactions between positively charged nanocarriers, polyethylene glycol-poly(l-lysine)-polymers, with negatively charged siRNA to facilitate delivery. MAPK1 siRNA/nanocarrier complex used in a model of glomerulonephritis demonstrated that MAPK1 suppression remarkably improved kidney function, reduced proteinuria, and ameliorated glomerular sclerosis. The expression of the profibrotic marker TGF-β was also significantly decreased after MAPK1 siRNA therapy and glomerulonephritis progression was prevented [49]. However, in contrast to AKI treatment, repeated siRNA administration will be necessary to achieve a therapeutic effect in chronic conditions. This necessity leads to some concerns for siRNA therapy, such as the medical cost and potential long-term side effects, which will be discussed later.

### Renal cell carcinoma

Advanced renal cell carcinoma (RCC) is highly resistant to standard chemotherapy regimens, resulting in a 5-year survival rate of only 10% for those with stage IV disease [50]. Gene therapy provides an efficient method for targeting the specific genes involved in RCC pathogenesis. For example, the HuR protein is a nucleocytoplasmic protein that plays an important role in the regulation of mRNA stability [51]. A recent study examined the effects of siRNA-mediated HuR gene silencing in multiple RCC cell lines *in vitro* and demonstrated a 60% reduction in tumor cell growth compared with control cells. These findings were then successfully replicated *in vivo* using tumor-bearing mice [52]. Shang *et, al.* further demonstrated the potential mechanism involved in the proliferative ability of RCC cells using siRNA technology [53]. Other siRNA experiments have revealed that histone H3 acetylation was responsible for preserving drug sensitivity in RCCs, which indicates the important role of epigenetics in RCC [54].

Nevertheless, silencing a single gene may cause tumor self-regulation and the development of drug resistance. Cancers, as well as other angiogenesis-related diseases, often result from the overexpression of multiple endogenous and exogenous pathogenic genes. One strategy for overcoming these challenges is using a cocktail therapy that combines multiple siRNAs targeting multiple disease-causing genes. Indeed, siRNA cocktails have demonstrated better anti-tumor potency compared with siRNAs that target a single gene [55,56] (Table 1).

**Table 1:** **Studies on kidney diseases using siRNAs**

**Investigators**	**Model**	**Modification**	**Transfection**	**Delivery**	**Target gene**	**Pathway**	**Reference**
Yang et al.	Porcine LLC-PK1	-	Cationic lipid	In vitro	Caspase-3	Apoptosis	[25]
Yang et al.	Porcine	-	UW solution	Ex vivo, renal artery injection and autologous blood perfusate	Caspase-3	Apoptosis	[22]
Yang et al.	Porcine	-	UW solution	In vivo, renal artery injection	Caspase-3	Apoptosis	[14]
Yang et al.	Porcine	LNA	UW solution	In vivo, renal artery injection and systemically iv.	Caspase-3	Apoptosis	[18]
Liu et al.	Mice	-	Plasmid	In vivo, hydrodynamic injection iv.	TLR9	Innate immunity	[48]
Hamar et al.	Mice	2’-O-ACE-RNA phosphoramidites	Phosphate buffered saline	In vivo, hydrodynamic injection iv.	Fas	Apoptosis	[28]
Wan et al.	Rats	-	Phosphate buffered saline	In vivo, renal artery injection	IKKβ	Inflammation	[27]
Xia et al.	Mice	-	Cationized gelatin microspheres	In vivo, injected via ureter	HSP47	collagen-producing, fibrosis	[29]
Molitoris et al.	Rats	2’O-methylation	Lipofectamine 2000	In vivo, iv.	p53	Apoptosis	[37]
Zheng et al.	Mice	-	Lipofectamine 2000	In vivo, hydrodynamic injection iv.	Complement 3 and caspases-3	Complement and apoptosis	[38]
Shimizu et al.	Mice	-	Nanocarrier complexation	In vivo, ip.	MAPK1	Immunity	[49]
Shang et al.	ACHN, A498 RCC cells	-	Lipofectamine 2000	In vitro	HIF-1α, HIF-2α	Tumorigenesis	[53]
Juengel et al.	Caki-1 RCC cells	-	Transfection reagent supplied by Qiagen	In vitro	HDAC1, HDAC2	Acetylation	[54]
Fujino et al.	Mice	-	Cationic lipid	In vivo, renal artery injection	p53	Apoptosis	[46]

UW: University of Wisconsin; TLR: toll-like receptor; siSTABLE: stability-enhanced siRNA; RCC: renal cell carcinoma; MAPK1: mitogen-activated protein kinase 1; HIF: hypoxia induced factor; HDAC: histone deacetylases.

## Off-target side effects/toxicities of siRNA

Off-target effects are one of the major obstacles for siRNA therapy. The induction of various side effects may be caused by unexpected perturbations between RNAi molecules and cellular components. The off-target effects of siRNA were first reported by Jackson and colleagues in 2003 [57]. Broadly speaking, off-target effects can be siRNA specific or non-specific. The former are caused by limited siRNA complementarity to non-targeted mRNAs. The latter, resulting in immune- and toxicity-related responses, are due to the construction of the siRNA sequence, its modification or the delivery vehicle.

The off-target effects associated with siRNA delivery fall into three broad categories: (1) microRNA-like off-target effects, referring to siRNA-induced sequence-dependent regulation of unintended transcripts through partial sequence complementarity to their 3′UTRs; (2) inflammatory responses through the activation of TLR triggered by siRNAs and/or delivery vehicles (such as cationic lipids and viruses); and (3) widespread effects on microRNA processing and function through the saturation of the endogenous RNAi machinery by exogenous siRNAs [58,59].

### MicroRNA-like off-target effects

The siRNAs and microRNAs share similar machinery downstream of their initial processing. Using several different siRNAs targeting the same gene, microarray profiling showed that each siRNA produced a unique, sequence-dependent signature. Sequence analysis of off-target transcripts revealed that the 3’ UTR regions of these transcripts were complementary to the 5’ end of the transfected siRNA guide strand [57]. It is now understood that for the off-targeting mechanism to occur, a perfect complementation between the nucleotide positions 2–7 or 2–8 (seed region) of the antisense strand and the 3’UTR of the transcript is necessary [58,60]. Base mismatches in the 5’ end of an siRNA guide strand reduced silencing of the original set of off-target transcripts, but introduced a new set of off-target transcripts with 3’ UTRs that were complementary to the mismatched guide strand [58].

RNAi regulation by miRNAs involves partial complementarity between the target RNA and miRNA. Because miRNAs cause gene silencing through mRNA degradation and translation inhibition, the siRNA mediated off-target effects may also be acting at two levels. For this reason, there should be greater emphasis on improving siRNA design, as well as monitoring gene and protein levels following RNAi therapy to account for any off-target effects.

### Recognition/Stimulation by the innate immune system

The recognition and stimulation of the immune system is a nonspecific off-target effect of siRNA therapy. The RNA-sensing pattern recognition receptors (PRRs), localized in endosomes, are the most important components of the innate immune system. The responses of PRRs to siRNAs are either TLR-mediated or non-TLR-mediated. The PRR responses are also associated with siRNA sequence specific side effects and have recently attracted lots of attentions from researchers [61]. RNA-sensing TLRs (TLR3 and TLR7) are predominantly located intracellularly and recognize nucleic acids released from invading pathogens. The non-TLR-mediated innate immune responses triggered by siRNA binding are linked to RNA-regulated expression of protein kinase (PKR) and retinoic acid inducible gene 1 (RIG1), which further induce caspase-3 and NF-κB expression, respectively. The activation of PRRs generates excessive cytokine release and subsequent inflammation [62].

Based on this second type of off-target RNAi effects, our group further investigated the mechanism of how short-acting caspase-3 siRNA impaired post-transplanted kidneys. The results suggested that the amplified inflammatory responses in caspase-3 siRNA preserved auto-transplant kidneys were associated with TLR3, TLR7 and PKR activation, which may be due to systemic compensative responses, although persistent actions initiated by short-acting caspase-3 siRNA cannot be completely excluded [63]. Other studies have also indicated that the horseshoe-like structure of TLR3 facilitates dsRNA recognition [64,65]. Interactions between TLR3 and dsRNA were originally reported in 2001 when TLR3-deficient mice exhibited reduced immune responses to dsRNA viruses [66].

Several studies have demonstrated that the immune response to siRNAs is cell type-dependent, due to the selective expression of TLRs. siRNAs stimulate monocytes and myeloid dendritic cells through TLR8 to produce pro-inflammatory cytokines, or activate plasmacytoid dendritic cells through TLR7 to produce type I interferons [67-69]. In addition, the volume of hydrodynamic naked siRNA delivery influences immune activation. Rácz et al. compared the immune responses induced by 50 μg siRNA dissolved in either low-volume (1 mL/mouse) or high-volume (10% of body weight, 2.5 mL/mouse in average) physiological salt solution delivered *in vivo*. Low-volume hydrodynamic injection induced slight alanine aminotransferase (ALT) elevation and mild hepatocyte injury, whereas high-volume hydrodynamic injection resulted in higher ALT levels and extensive hepatocyte necrosis. High-volume hydrodynamic injection also led to a time-dependent slight increase in IFN-related gene expression [70]. Collectively, these studies suggest that there is a need for improving siRNA design, establishing experimental controls and carefully interpreting results.

### Systemic compensative actions post siRNA treatment

Silencing the target mRNA occasionally induces systemic compensative actions *in vivo*. In our previous research, the unmodified naked caspases-3 siRNAs aggravated renal graft injury [14]. A potential reason for this effect is due to the kidney structure. The pore size of the glomerular filtration barrier is approximately 8 nm and naked siRNA has been observed to pass through this barrier into the urine [20]. Upon reperfusion, the caspases-3 siRNA in the preserved kidney was flushed, while the local siRNA in the post-CS kidneys may also rapidly disappear due to a surge of blood flow into the kidney, leading to siRNA degradation and elimination. After transplantation, a series of *in vivo* complementary responses to the lower level of caspase-3 mRNA in the post-CS kidneys was initiated, which first led to an increase in caspase-3 mRNA synthesis. As the consequence of siRNA degeneration and caspase-3 mRNA synthesis, the level of caspase-3 mRNA increased in the siRNA preserved post-transplant kidneys. It has been suggested that a post-transplant feedback loop was initiated by the effective delivery of caspase-3 siRNA during CS, which led to increased mRNA synthesis after transplantation. However, with improved nuclease stability, the LNA modified caspases-3 siRNA protected both the post-CS and post-transplant renal grafts. More importantly, the modified siRNAs did not induce systemic compensative actions, which was proven by monitoring systemic inflammation [18].

## From bench to bedside: clinical trials

The numbers of RNAi-based preclinical studies and clinical trials have grown over the past several years. To date, there have been 27 registered clinical trials using siRNA worldwide. These studies include retinal degeneration, dominantly inherited brain and skin diseases, viral infections, respiratory disorders, metabolic diseases and, of particular note, kidney diseases. In 2011, Quark Pharmaceuticals completed a phase I, randomized, double-blind, dose escalation, safety and pharmacokinetic study (NCT00554359) on QPI-1002, also designated I5NP, which was a synthetic siRNA that temporarily inhibits p53 expression that is in early development for acute kidney failure therapy. I5NP is the first siRNA to be systemically administered in humans. Based on data from animal studies, the intravenous injection in the human studies was performed within 4 hours of bypass surgery and pharmacokinetic data were obtained during the first 24 hours. Follow-up was conducted for safety and dose-limiting toxicities until hospital discharge and then by phone 6–12 months after surgery. Recently, Quark initiated a subsequent clinical trial to determine whether a single administration of I5NP can prevent delayed graft function in kidney transplant recipients. Data from this study will be used to identify I5NP doses for follow-on efficacy studies (NCT00802347). Another ongoing phase I trial investigating solid tumors, including RCCs, was conducted by Calando Pharmaceuticals. The investigators used CALAA-01, whose active ingredient is a type of siRNA, to inhibit tumor growth and/or reduce tumor size. This siRNA inhibits the expression of the M2 subunit of ribonucleotide reductase and resists nuclease degradation by using a stabilized nanoparticle that targets tumor cells (NCT00689065, the above clinical trials can be found at ClinicalTrials.gov, Table 2).

**Table 2:** **Clinical trials of siRNA therapy in kidney diseases**

**Study**	**Target/siRNA drug**	**Status**	**Disease**
NCT00554359	I5NP	Phase I, completed	Kidney injury; Acute renal failure
NCT00802347	I5NP	Phase I/II, active, not recruiting	Delayed graft function in kidney transplantation
NCT00689065	M2 subunit of ribonucleotide reductase/CALAA-01	Phase I, terminated	Solid tumor cancers
NCT02166255	siRNA-transfected peripheral blood mononuclear cells APN401	Phase I, not yet recruiting	Melanoma, kidney cancer, pancreatic cancer, or other solid tumors that are metastatic or cannot be removed by surgery

## Perspectives and challenges

Despite the enormous potential of siRNA therapy, additional research must be performed before its large-scale clinical application.

### Target gene selection

Genome-wide or pathway-specific siRNA libraries have become available using high-throughput screening approaches. Establishing *in vitro* pre-screening leads to signaling pathway prediction and target validation in *in vivo* renal disease. However, choosing one or a set of reasonable target gene(s) is the key for designing specific siRNA treatments. The pathophysiological changes during kidney disease, like any other disease, refer to a complex gene and protein regulation network. For example, the network that exists during kidney transplantation involves the original conditions of the donors and the interactions between the donor kidneys and the recipients, which could direct the progression, as well as the recovery, of the injury. Fortunately, transcriptome measurements of the transplant kidney may provide a comprehensive understanding of gene regulation and would be beneficial for target gene selection.

**Figure 2:**
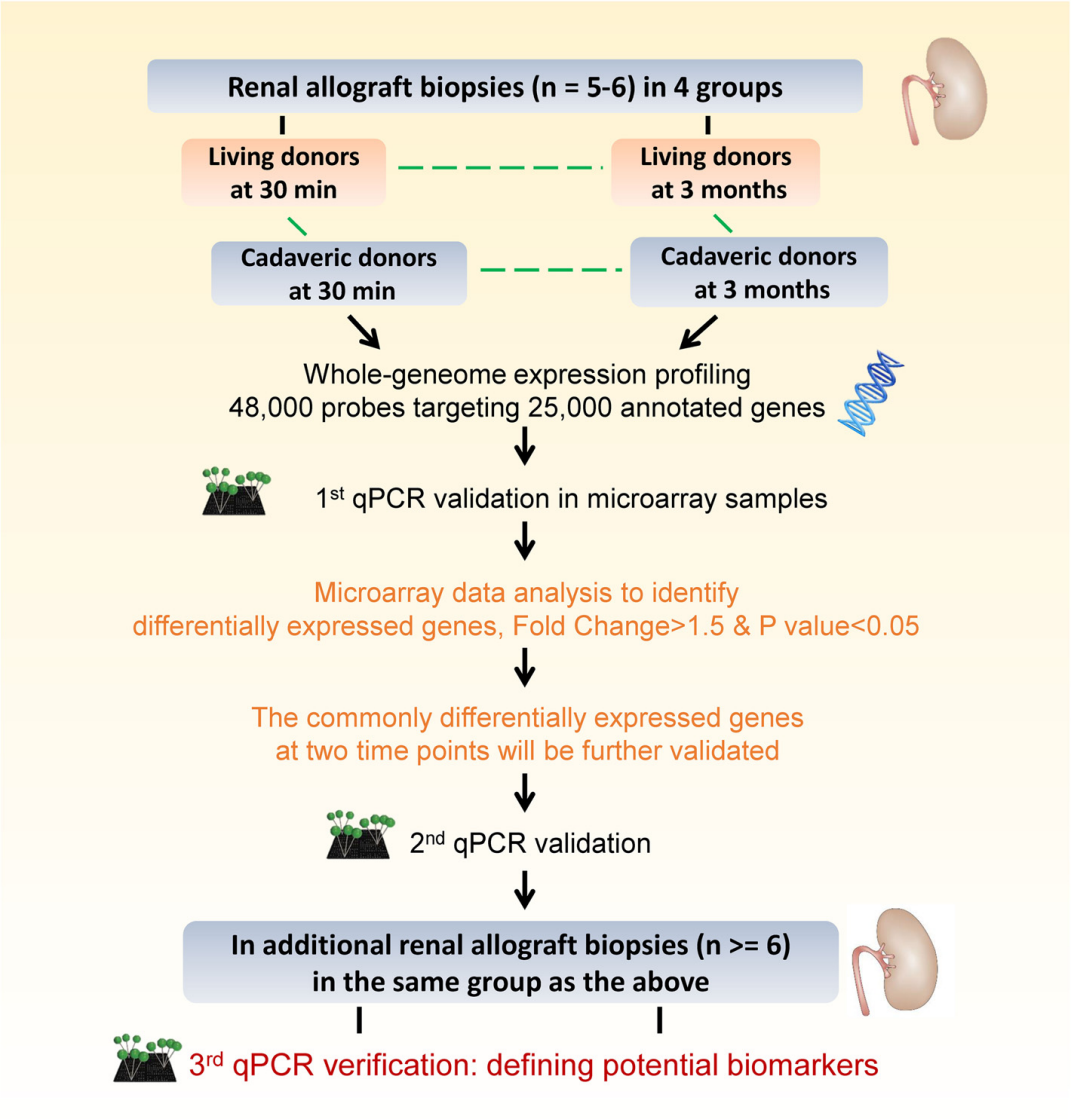
**Study design of identifying and validating potential targets for siRNA treatment using human transplant kidney biopsies.**

Mueller et al. analyzed the transcriptome of post-reperfusion implant biopsies in living donors (LD) and deceased donors (DD). Hundreds of mRNAs were identified that predicted delayed graft function [71]. In a recent prospective study using human post-transplant kidney biopsies, 20 mRNAs and two miRNAs were identified as molecular signatures of AKI. Elevated secretory leukocyte peptidase inhibitor in AKI allografts was validated and miR-182-5p was identified as a molecular regulator [72]. These genes could be used as potential targets of siRNA therapy. We recently identified 3 times more differentially expressed genes in renal allograft biopsies between living donors and cadaveric donors at 30 min than 3 months post-transplantation. The majority of these differentially expressed genes are responsible for acute responses at 30 min, but involved in inflammation, nephrotoxicity and proliferation at 3 months (Figure 2). These divergent transcriptome signatures between two types of donors might be linked with not only the initial injury of the donors, but also the immune responses of the recipients.

Another method for selecting target genes is by identifying their translation product proteins. To find a single or a set of crucial protein(s) involved in kidney allograft rejection, we explored potential transcriptional factors and regulation networks in 352 kidney transplant recipients, of which 85 suffered from acute rejection (AR). The results demonstrated that the dominant processes and responses were associated with inflammation and complement activation in AR. A number of transcription factors were identified in AR patients, including NF-κB, signal transducer and activator of transcription (STAT) 1 and STAT3 [73]. Our recent study further revealed inflammation-derived kidney allograft injury, such as AR, chronic rejection, and impaired renal function without rejection. We identified 12 common proteins and 11 level-specific proteins from the phenotype-related protein–protein interaction networks [74]. These potential biomarkers also provide valuable targets for transplant-related injury siRNA design.

### Timely application

Compared with shRNA, an advantage of siRNA for AKI therapy is the time-controlled treatment. Silencing the target gene for a short time or a long time should be addressed before RNAi application. The silenced genes may be multifunctional according to the surrounding milieu. For example, caspase-3, generally considered an executor in cell apoptosis, should be inhibited in tissue injuries. However, it is also a loyal scavenger in malignant transformation cells, which could be an unavoidable side effect in any caspase-3-targeting siRNA therapy. For AKI, siRNA ineffectiveness is needed after the therapeutic time window. Additionally, siRNA application avoids intracellular traffic. In certain circumstances, shRNA delivery could be harmful to the organ or even fatal. A study from Grimm et al. investigated the long-term effects of sustained high-level shRNA expression in the livers of adult mice. An evaluation of 49 distinct adeno-associated virus/shRNA vectors, with unique lengths and sequences that were directed against six targets, showed that 36 vectors resulted in dose-dependent liver injury, with 23 ultimately causing death. The observed morbidity was associated with the downregulation of liver-derived microRNAs (miRNAs), indicating possible competition of the latter with shRNAs (*through saturation of the endogenous RNAi machinery by the exogenous siRNAs*) for the limited cellular factors required for the processing of various small RNAs [75]. Therefore, controlling intracellular shRNA expression levels will be imperative, but siRNA would not influence the endogenous process of RNA degradation mediated by miRNAs.

### Cell-specific delivery

For optimized RNAi therapy, it should be determined whether regional delivery with partial knockdown or systemic delivery with global knockdown is required. However, there is still a lack of target specificity during systemic siRNA delivery.

It is known that p53 in PTCs promotes AKI, whereas p53 in other tubular cells does not [40]. Therefore, the design of PTC-specific p53 siRNA is necessary. It is also expected that, for example, apoptosis-inducing siRNA should be directly delivered into tumor cells, rather than the surrounding normal cells.

Recently, antibody conjugation technology has made tumor-targeting drug delivery systems available. In general, these systems consist of a tumor recognition moiety and a cytotoxic warhead connected directly or through a suitable linker to form a conjugate. The conjugate can be regarded as a “guided molecular missile” that specifically targets tumor unique antigens [76]. Inspired by cancer therapy strategies, siRNAs have also been “packed” to be delivered to target organs, even cells. Recently, a type of asymmetric liposome particle (ALP) has been developed, which highly efficiently encapsulates siRNA without nonspecific cell penetration. Two types of lipid inverted micelles have been prepared for the purpose of obtaining asymmetric liposome particles. The ALPs protected siRNA from ribonuclease degradation. ALPs without any surface modification elicited almost no uptake into cells, while the polyarginine peptide surface-modified ALPs induced nonspecific cell penetration. The conjugation of an anti-human epidermal growth factor receptor antibody (anti-EGFR) to the ALPs induced EGFR-mediated uptake into non-small cell lung cancer cell lines, but not into NIH-3 T3 cells that do not have the receptor [77]. This result represents great progress in siRNA delivery system development. Antibody-mediated specific recognition may be more mainstream than virus-mediated recognition in the future.

Nanoparticulate systems have emerged in last few years as an alternative material for advanced diagnostic and therapeutic applications in medicine. Compared with molecular medicine, nanotechnology offers many advantages that overcome the range of challenges and barriers summarized in the previous section, particularly the bioavailability and biodistribution of therapeutic agents. The first remarkable property of nanoparticles is their superior *in vivo* retention due to decreased enzymatic degradation and sequestration by phagocytes in the reticulo-endothelial system. This property is primarily attributed to their immunochemically inert surfaces that are in contact with the biological environment. Methods of conjugating siRNAs with other inert and biocompatible molecules, such as cholesterol and long-chain fatty acids, have also been reported [78,79].

### Minimizing side effects

The knockdown of two or more genes simultaneously using an siRNA cocktail has been recently reported. Many applications of siRNA cocktails have demonstrated significant benefits compared with siRNA targeted to a single gene, particularly in anti-cancer and anti-viral therapy [56,80]. A high concentration of individual siRNAs may represent the key off-target effect in terms of competition for endogenous miRNA biogenesis machinery. Therefore, the other advantage of an siRNA cocktail is the relatively low concentration of each siRNA, which may reduce off-target signatures without sacrificing silencing potency [55].

## Conclusions

The kidney is a comparatively easy target organ for siRNA therapy due to its unique structural and functional characteristics. siRNA intervention is effective, feasible and has great potential for fighting against kidney diseases. The safety of siRNA therapy has been proven by rapidly emerging clinical studies and off-target and compensative responses can be managed using several strategies. We believe that optimized siRNA therapy, in conjunction with advanced genetic screening technologies, could facilitate timely and specific treatment for kidney diseases, as well as other organ diseases in the near future.
